# Act Locally, Act Globally—Microbiota, Barriers, and Cytokines in Atherosclerosis

**DOI:** 10.3390/cells10020348

**Published:** 2021-02-07

**Authors:** Natalia Kurilenko, Aliia R. Fatkhullina, Aleksandra Mazitova, Ekaterina K. Koltsova

**Affiliations:** 1 Department of Medicine and Department of Biomedical Sciences, Cedars-Sinai Medical Center, 8700 Beverly Blvd, Los Angeles, CA 90048, USA; Natalia.Kurilenko@cshs.org (N.K.); Aleksandra.Mazitova@cshs.org (A.M.); 2 Department of Pathology, University of Chicago, Chicago, IL 60637, USA; fatkhullina@bsd.uchicago.edu

**Keywords:** cytokines, atherosclerosis, microbiota, barrier tissue, inflammation

## Abstract

Atherosclerosis is a lipid-driven chronic inflammatory disease that is characterized by the formation and progressive growth of atherosclerotic plaques in the wall of arteries. Atherosclerosis is a major predisposing factor for stroke and heart attack. Various immune-mediated mechanisms are implicated in the disease initiation and progression. Cytokines are key mediators of the crosstalk between innate and adaptive immune cells as well as non-hematopoietic cells in the aortic wall and are emerging players in the regulation of atherosclerosis. Progression of atherosclerosis is always associated with increased local and systemic levels of pro-inflammatory cytokines. The role of cytokines within atherosclerotic plaque has been extensively investigated; however, the cell-specific role of cytokine signaling, particularly the role of cytokines in the regulation of barrier tissues tightly associated with microbiota in the context of cardiovascular diseases has only recently come to light. Here, we summarize the knowledge about the function of cytokines at mucosal barriers and the interplay between cytokines, barriers, and microbiota and discuss their known and potential implications for atherosclerosis development.

## 1. Introduction

Cytokines are critical mediators of immune and inflammatory responses and tissue homeostasis in health and disease. Atherosclerosis is the most prevalent form of cardiovascular disease (CVD), which accounts for nearly 18 million deaths annually, making it a number one cause of death worldwide [1]. Atherosclerosis is a lipid-driven chronic inflammatory disease; and atherosclerotic plaques are infiltrated by all major immune cell subtypes. Consequently, cytokines have been shown to regulate inflammatory milieu in atherosclerosis, both locally (in the plaque) and systemically. Genetic or pharmacological studies in mice and humans defined the contribution of cytokines and their receptors to the pathogenesis of atherosclerosis [2] to the most extent by their suggested action inside the plaque, i.e., locally. However, cell-type specificity of cytokine signaling as well as possible roles of cytokines at distant organ sites and barrier surfaces in the regulation of CVD have recently got an increased attention. Cytokine expression is readily induced by microbe-derived, environment-driven, or stress-induced stimuli, and cytokines are essential for the regulation of antimicrobial immune responses as well as various metabolic responses [3,4,5,6,7,8]. Coupled with the recent discoveries of the emerging roles of microbiota and microbiota-derived metabolites in CVD development and progression, it begs for further in-depth (re)-evaluation of local versus distant actions of cytokines in atherosclerosis and the complex interaction of cytokine signaling with microbiota-derived and metabolic cues. With the exceptions of overt infections, a great deal of host–microbiota interactions relevant for tonic and chronic stimulation for inflammatory and metabolic processes happen at the barrier surfaces, such as gastrointestinal and oral epithelia. In this review, we concentrate on how a diverse array of cytokines plays essential roles in regulation of CVD development, with a special focus on common mechanisms that place these mediators of inflammation upstream and downstream from the host–microbiota interactions at the barrier surfaces and additional contributions of distant cytokine-driven processes to the pathogenesis of CVD. While various cytokines have been shown to regulate atherosclerosis [2], here we focus on cytokines which have an established role in atherosclerosis and are implicated into the regulation of barrier tissue homeostasis.

## 2. The Role of Selected Cytokines in Atherosclerosis

### 2.1. Cytokines of the Interleukin (IL)-1 Family in Atherosclerosis

The IL-1 cytokine family consists of 11 members, including IL-1α, IL-1β, IL-18, IL-33, members of the IL-36 sub-family (α, β, γ), and IL-37 [9]. IL-1α and IL-1β, founding members of the family, are typically produced by myeloid cells as well as some stromal and epithelial cells and exert their physiological roles via IL-1 receptor (IL-1R) complex expressed on many different cells of immune system as well as non-immune cells [9]. As both IL-1α and IL-1β cytokines were found to be elevated in atherosclerotic plaques [10,11], their contribution to atherosclerosis development has been extensively investigated during the past decade [12,13,14,15,16,17,18,19,20]. IL-1α has been shown to promote atherosclerosis development as both atherosclerosis-prone apolipoprotein E (*Apoe*)- and low-density lipoprotein receptor (*Ldlr*)-deficient mice reconstituted with IL-1α-deficient bone marrow demonstrated a significant reduction of atherosclerotic lesions [17,19]. Whole-body genetic ablation of IL-1β in *Apoe*^−/−^ mice significantly decreased atherosclerotic lesion size [20]. Consistent with that, *Ldlr*^−/−^ mice with hematopoietic deficiency of IL-1α and IL-1β were also protected from the disease development [16]. Moreover, anti-IL-1β monoclonal antibody reduced atherosclerotic plaque formation in *Apoe*^−/−^ mice, suppressing the production of key pro-atherogenic cytokines IL-6, TNFα, and chemokine CCL2 [13]. Global blockade of IL-1R signaling in mice by genetic knockout of IL-1R or administration of soluble IL-1R antagonist (anakinra) also significantly reduced atherosclerotic lesion size [14,21]. All these findings suggested that IL-1β could serve as a promising therapeutic target for the reduction of cardiovascular disease risk. The recent Canakinumab Anti-inflammatory Thrombosis Outcome Study (CANTOS) trial revealed that anti-IL-1β antibody (canakinumab) decreases the risk of recurrent cardiovascular events in high-risk patients with a myocardial infarction history [22]. This seems to have a high biological significance as it was observed in a high-risk group of patients independent of their lipid levels [22]. On the other hand, IL-1β neutralization during the late stages of atherosclerosis did not yield a significant reduction in disease manifestations in a mouse model [18]. More recent work implied IL-1 as one of the prominent regulators of vascular smooth muscle cells [12], as IL-1R ablation in smooth muscle cells reduced atherosclerotic plaque size [18], suggesting a cell-specific role of this cytokine, which could vary at different disease stages.

In the context of atherosclerosis, IL-18 expression is predominantly detected in macrophages, while IL-18R is expressed on endothelial cells (ECs), smooth muscle cells (SMCs), lymphocytes and macrophages [23,24]. Increased expression of IL-18 was found in human atherosclerotic plaques and was associated with signs of plaque instability [24]. However, whether serum levels of IL-18 could serve as a predictor of major cardiovascular events is still debatable [25]. Other models of inflammation suggest that IL-18 is a potent inducer of IFNγ, another prominent pro-atherogenic cytokine [2]. Indeed, recombinant IL-18 administration to *Apoe*^−/−^ mice promoted atherosclerosis development in an IFNγ-dependent manner [26], while its neutralization by the natural inhibitor IL-18 binding protein (IL-18BP) [27] or genetic knockout of IL-18 significantly reduced disease progression [28]. IL-18 can trigger atherosclerosis-relevant IFNγ expression even in the absence of T cells, as the administration of recombinant IL-18 to lymphocyte-deficient SCID mice led to increased lesion size accompanied by elevated IFNγ levels in the circulation [29]. Recent studies have revealed that IL-18 may affect atherogenesis via signals through both IL-18R and the Na-Cl co-transporter (NCC), which co-localizes with IL-18R in atherosclerotic lesions. Only a combined deficiency of *Il18r* and the NCC but not the single deficiency of either protein resulted in ameliorated atherosclerosis in *Apoe*^−/−^ mice [30]. Moreover, IL-18 is also important for quality control of microbiota and taming of inflammation-regulated metabolic diseases, all of which immensely impact atherosclerosis and whose modeling requires precise controls in long-term in vivo experiments [31,32,33].

### 2.2. Cytokines of the IL-17 Family in Atherosclerosis

The IL-17 family includes six members, IL-17A, IL-17B, IL-17C, IL-17D, IL-17E (also known as IL-25), and IL-17F, which play a crucial role in autoimmunity, cancer, and other inflammatory conditions [34]. In the context of atherosclerosis, IL-17A is the most well-studied member of this cytokine family. IL-17A is produced by CD4^+^ T helper (Th) 17, type 3 innate lymphoid cells (ILC3), and γδT cells, with some context-dependent contribution of natural killer T (NKT) and CD8^+^ (Tc17) cells [34]. IL-17A expression and lineage identity of IL-17-producing cells require a transcription factor, the retinoic acid receptor-related orphan receptor gamma t (RORγt), whose induction and stability of expression are facilitated by the action of IL-1, IL-23, and IL-6 cytokines [35]. The differentiation of Th17 cells is dependent on IL-1 signaling as well as IL-23. Recent work also revealed that many of the IL-17A-expressing cells are also capable of producing other cytokines, for example, IL-17F and IL-22. IL-17A and IL-17F can act as homodimers or as an IL-17A/IL-17F heterodimer, and many of the IL-17F functions are similar to those of IL-17A. IL-17A, IL-17F, and their heterodimer signal through the same receptor with decreasing affinity to ligand in the order IL-17A > IL-17A/IL-17AF > IL-17F [34]. The IL-17A receptor is a heterodimer of IL-17RA and IL-17RC subunits. IL-17RA and IL-17RC are abundantly expressed in many tissues [36,37]. Recently, IL-17RD, together with IL-17RA, was also reported to bind IL-17A [38]. This signaling was shown to play an important role in IL-17-mediated skin inflammation [38], but its role in other physiological settings requires further investigation.

The role of IL-17A in atherosclerosis has been extensively investigated [39,40,41,42,43,44,45,46,47,48,49]; however, it still remains debatable. Several groups have reported the presence and accumulation of IL-17A-producing cells during atherosclerosis progression [45,48]. However, genetic studies have revealed contradicting results. Acceleration of the disease was observed in *Il17a*^−/−^*Apoe*^−/−^ mice [42]. Moreover, mice with suppressor of cytokine signaling 3 (SOCS3) deficiency, characterized by elevated IL-17A and reduced IL-10 expression, showed ameliorated disease [46]. Neutralization of IL-17A in SOCS3-deficient mice enhanced atherosclerosis development associated with T cell accumulation in the aorta and elevated vascular cell adhesion molecule 1 (VCAM-1) expression [46]. Moreover, administration of recombinant IL-17 to *Ldlr*^−/−^ mice resulted in the amelioration of the disease associated with reduced CD3^+^ T cell accumulation and VCAM-1 expression in the aorta [46]. A recent study revealed that mice transplanted with Tri-partite motif 21 (TRIM21)-deficient bone marrow demonstrated atherosclerotic plaques with a more stable phenotype, characterized by a higher collagen content and a thicker fibrous cap [39]. This phenotype was associated with *Trim21*-deficiency-driven Th17 responses and IL-17A production in the vessel wall, suggesting a possible role for IL-17 in plaque stability and therefore arguing for an atheroprotective role of this cytokine [39]. A similar phenotype was observed in mice transplanted with bone marrow from T-cell-specific knockout of *Smad7*, where also elevated expression of IL-17A was observed [44]. Neutralization of IL-17A in these mice prevented increased fibrous cap formation and reduced collagen levels, suggesting the role of IL-17A in plaque stabilization [44]. Several studies have reported a pro-atherogenic role of IL-17A [40,43,45,47,48,49], with a strong reduction of atherosclerotic lesion size in *Il17a*^−/−^*Apoe*^−/−^ and *Il17ra*^−/−^*Apoe*^−/−^ mice, where the atheroprotective effect of IL-17A ablation was associated with reduced macrophage accumulation [40]. Neutralization of IL-17 with the adenovirus-encoded soluble IL-17RA inhibitor or monoclonal antibodies also resulted in disease attenuation [43,49]. IL-17C, produced by vascular smooth muscle cells, has been suggested to play a pro-atherogenic role, affecting the recruitment of various pro-inflammatory cells to the aorta [50]. It is important to note that anti-IL-17 treatment in psoriasis, which is a prominent risk factor for cardiovascular diseases [51,52], strongly reduced the psoriasis burden; however, the effect on atherosclerosis was rather mild [53]. The discrepancy of the observed roles of IL-17 and IL-17 signaling in atherosclerosis could be potentially explained by the unique role of IL-17R in different cell types, inviting further investigations of cell-type-specific roles of this cytokine in the context of atherosclerosis.

### 2.3. Cytokines of the IL-10 Family in Atherosclerosis

The IL-10 family consists of nine members, i.e., IL-10; IL-20 subfamily members IL-19, IL-20, IL-22, IL-24, and IL-26; and IL-28A, IL-28B, and IL-29 cytokines, which are also known as type III interferons (IFNs) [54]. To date, the contribution to atherosclerosis has been addressed for IL-10, IL-19, and IL-22.

IL-10 is a immunoregulatory cytokine produced mostly by immune cells, including T cells, B cells, dendritic cells (DCs), macrophages, and natural killer (NK) cells [54]. The IL-10 receptor is expressed in most hematopoietic cells as well as on several non-hematopoietic cells such as colonic epithelial cells and fibroblasts [55]. IL-10 expression was detected in advanced human atherosclerotic plaques, and its expression was associated with reduced inducible nitric oxide synthase expression and cell death [56]. Animal studies showed that IL-10-deficient mice developed significantly more atherosclerosis [57,58,59], with elevated T cell accumulation, IFNγ expression, and reduced collagen content in the lesion [57], concordant with its role as a potent inhibitor of inflammatory responses. The overexpression of IL-10 and its administration suppressed the disease development, implying the protective role of this cytokine in atherosclerosis [57,58,60,61]. More recent studies revealed the specificity of the IL-10 cellular source, demonstrating the importance of T-cell-derived but not B-cell-derived IL-10 in taming the severity of atherosclerosis [61,62,63].

The expression of IL-22, similarly to IL-17A, is regulated by IL-1 and IL-23 and originates from activated Th17/Th22 and ILC3 in an RORγt- and aryl hydrocarbon receptor (AHR)-dependent manner [64]. While IL-1 and IL-23 are often induced by microbial signals, the involvement of RORγt and AHR makes IL-22 expression modifiable by metabolic (sterol metabolites) and environmental (endogenous and exogenous AHR ligands) cues. The IL-22 receptor (IL-22R) is expressed mostly in non-hematopoietic cells, such as fibroblasts, adipocytes, and hepatocytes, as well as epithelial cells, including keratinocytes and mucosal epithelial cells [65]. In the intestine, IL-22 is crucial in the maintenance of mucosal barrier integrity and commensal microbiota via control of barrier peptides and mucus production, epithelial cell proliferation, and host defense to extracellular pathogens [66,67,68]. IL-22 signaling is shown to suppress metabolic disorders, as IL-22 administration to obese *db/db* leptin receptor-deficient mice reversed many of the observed metabolic symptoms [69]. Moreover, IL-22 was implicated into the regulation of lipid metabolism in the liver and adipose tissue [69].

The role of IL-22 in atherosclerosis has been addressed in a few recent studies. Genetic ablation of IL-22 in *Apoe*^−/−^ mice showed a small reduction in lesion size [70]. On the other hand, ablation of IL-22 in hematopoietic cells heightened atherosclerosis in a mouse model, while administration of an IL-22-Ig-stabilized recombinant cytokine suppressed the disease development in *Ldlr*^−/−^ and IL-23-deficient *Ldlr*^−/−^ animals [71]. The latter study further linked the progression of atherosclerosis with impaired function of intestinal epithelial cells and expansion of pathogenic and pro-atherogenic microbiota that contributed to the disease pathogenesis.

### 2.4. Cytokines of the IL-6/IL-12 Superfamily in Atherosclerosis

The IL-6/IL-12 superfamily consists of IL-6 family members (IL-6, IL-11, leukemia inhibitory factor (LIF), ciliary neurotrophic factor (CNTF), neuropoetin (NP), cardiotrophin-1 (CT-1), cardiotrophin-like cytokine (CLC), and oncostatin M (OSM)) and IL-12 family members (IL-12, IL-23, IL-27, IL-35, and IL-39) [72]. Our review is focused on IL-6, IL-12, and IL-23 members of the family since their role in the regulation of barrier tissue homeostasis has been investigated.

IL-6 is produced by a variety of stromal and immune cells. The receptor for IL-6 consists of IL-6R, which is expressed by hepatocytes, various epithelial cells and some immune cells, and the universally expressed signal-transducing subunit β also known as gp130 [73]. Cleaved from the cell surface, IL-6R could capture IL-6 and bind to membrane-bound gp130 and activate it, transmitting IL-6 signals to cell types that are typically devoid of IL-6R expression and expressing only gp130 as part of the so-called IL-6 trans-signaling [73].

Elevated expression of IL-6 was suggested to be a risk factor for coronary artery disease [74]. Given the fact that IL-6 is readily induced in almost any inflammatory condition, it is still not clear whether this is a causative or a correlative effect. Animal model studies came to controversial conclusions, implying both atherogenic and atheroprotective function for this cytokine. Early work showed that treatment with recombinant IL-6 dramatically increased the lesion size in *Apoe*^−/−^ mice [75]. Whole-body IL-6 deficiency suggests its atheroprotective role, since *Apoe*^−/−^*Il6*^−/−^ mice were characterized by increased atherosclerotic lesions with reduced collagen content and IL-10 production, but immune cell recruitment into atherosclerotic plaque was decreased [76]. Conversely, another study revealed that 1-year-old chow-diet-fed *Apoe*^−/−^*Il6*^−/−^ mice had an increased lesion size, whereas 16-week-old chow-diet-fed *Apoe*^−/−^*Il6*^−/−^ mice had no significant difference in lesion size compared to *Apoe*^−/−^*Il6*^+/−^ and *Apoe*^−/−^*Il6*^+/+^ controls [77].

Later, it was demonstrated that the type of IL-6/IL-6R signaling most likely would differently influence disease development. Membrane-bound IL-6R (this type of IL-6 signaling is known as “classical” signaling) was shown to exert anti-inflammatory and tissue regenerating effects, while binding of IL-6 to soluble IL-6R (sIL-6R) (“trans-signaling”) mediates pro-inflammatory responses via gp130 [78], perhaps in different target cell types, in comparison with signals from membrane-bound IL-6R. Inhibition of IL-6 trans-signaling with soluble glycoprotein 130 (sgp130Fc) protein led to a significant reduction in atherosclerosis in *Ldlr*^−/−^ mice, with reduced macrophage infiltration to the plaques [79]. These data are indicative of the additional need to investigate the cell-specific role of IL-6 in atherosclerosis, taking into consideration possible types of IL-6R signaling.

IL-12 is produced by dendritic cells and macrophages and consists of two subunits, IL-12p35 and IL-12p40 (shared with IL-23), with the receptor being expressed mostly by activated T and NK cells [80]. Recombinant IL-12 administration aggravated atherosclerosis [81], while IL-12 deficiency in *Apoe*^−/−^ mice significantly reduced the atherosclerotic lesion size and macrophage accumulation in the aorta [82], suggesting a pro-atherogenic role for this cytokine. However, due to the high combinatorial ability between cytokines of this family and the shared subunit with IL-23, the specificity of the effects described in earlier studies is not completely clear; therefore, additional work addressing the cell-specific role of IL-12R signaling in the context of atherosclerosis could be of great interest.

IL-23 is a heterodimer of IL-23p19 (unique) and IL-12p40 (shared with IL-12) subunits. IL-23 is a well-established regulator of IL-17A and IL-22 inflammatory cytokine production by CD4^+^ T helper IL-17-producing (Th17) cells, γδT cells, and type 3 innate lymphoid cells (ILC3) [83]. The IL-23–IL-17 cytokine signaling axis drives several chronic inflammatory diseases, including multiple sclerosis (MS), arthritis, psoriasis, and inflammatory bowel disease (IBD); and genetic inactivation or pharmacological blockade of IL-23 signaling blocks the disease development. Increased IL-23 expression in animals and patients with developed atherosclerosis has been demonstrated, suggesting a pro-inflammatory and pro-atherogenic role of this cytokine [84]. However, genetic ablation of IL-23 or IL-23R in hematopoietic cells unexpectedly accelerates atherosclerosis development, particularly due to the loss of downstream IL-22 expression and IL-22-dependent intestinal homeostasis and host–microbiota détente [71]. Enforced IL-22 signaling by administration of recombinant protein rectified IL-23/IL-23R deficiency and ameliorated atherosclerosis [71]. Interestingly, side effects arising from targeting IL-12 and IL-23 have been reported in a subset of patients in clinical trials, with most of these being major adverse cardiovascular events (MACE) [85], with a higher incidence of these effects in people with elevated cardiovascular risk factors [86]. These data suggest that despite elevated expression of IL-23 in atherosclerosis, its global neutralization may not be always beneficial for patients with CVD.

Potential phenotypic discrepancies with regard to the role of cytokines and inflammatory mediators observed in different labs, models, and animal facilities, along with the very high variability and diversity in the magnitude of cytokine induction and signaling (Table 1), as well as disease severity and manifestations in human patients, may point, among other things, to the environmental component driving inflammation in atherosclerosis. Such environmental component may be remarkably diverse and modifiable, subject to lifestyle-dependent changes, and in part may be mechanistically driven by a collaboration of dietary content and commensal microbiota, which interact with the host, primarily at barrier surfaces.

## 3. Mucosal Barriers as Gatekeepers for Microbiota: Potential Implication for Atherosclerosis

**Figure 1:**
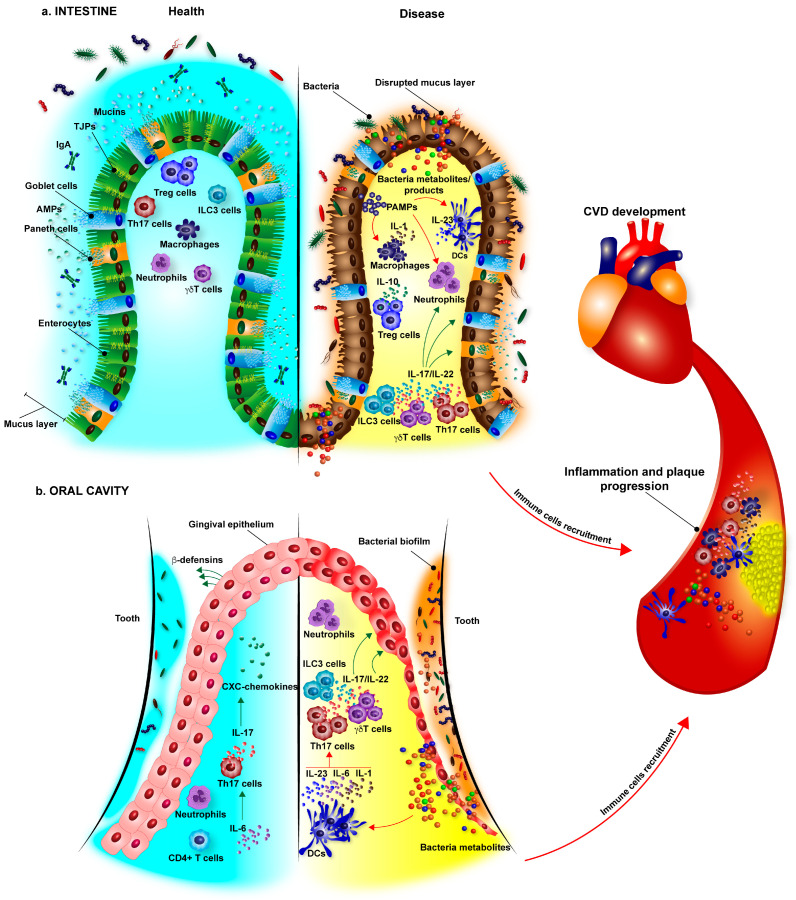
Mucosal barrier in health and disease: implication to atherosclerosis. (**a**) The barrier in the intestine consists of the intestinal epithelium; a mucus layer, which is composed of mucins and other structural proteins; antimicrobial peptides (AMPs) and IgA molecules, which bind to microbes and reduce their mobility in the mucus; and resident and infiltrating immune cells. Left. The function of intestinal epithelial cells, tight junction proteins (TJPs), antimicrobial peptide expression, and IgA translocation is tightly regulated by cytokine signaling that is important for the control of intestinal permeability and bacteria or bacteria-derived product translocation. A large number of immune cells (Treg cells, neutrophils, macrophages, Th17 cells, γδT cells, ILC3) are present in the normal gut lamina propria to maintain tissue integrity and produce cytokines, which regulate the anti-microbial response. Reciprocal regulation between microbiota and cytokines, i.e., the microbiota composition controls the production of cytokines, while cytokines regulate intestinal epithelial cells and infiltrating immune cells, thereby keeping microbiota in check, is crucial for the maintenance of a healthy barrier. Right. Unhealthy diet, chronic inflammation, infections, or altered cytokine signaling could lead to dysbiosis and increased intestinal permeability. This could trigger local or systemic (via circulation bacterial products or metabolites) activation of immune cells. Immune cells, including Th17, γδT, ILC3, neutrophils, and monocytes/macrophages, could migrate to the circulation or produce inflammatory mediators that could reach atherosclerotic plaque to promote inflammation. Alternatively or in addition, microbial products (i.e., LPS) or metabolites could promote activation of immune cells in the intestine, in the circulation, or in atherosclerotic plaque. (**b**) The barrier in the oral cavity is represented by squamous epithelial cells, saliva enriched with antimicrobial peptides, IgA, and infiltrating immune cells. Junctions between epithelial cells, antimicrobial peptide production, and immune cell activation are tightly regulated by cytokine signaling. Left. Normal barrier function prevents the expansion of pathogenic bacteria and their translocation to the tissue and circulation. Th17 cells and neutrophils play an important role in host defense at the health gingival barrier. Right. Inflammatory disorders such as infections or physiological damage of the epithelium cause increased translocation of bacteria, bacteria-derived metabolites, or bacterial products to the tissue and systemic circulation. This contributes to immune cell activation, which could migrate to other tissues such as aorta, where they, together with other factors, aggravate an inflammatory response. CVD—cardiovascular disease; IL—interleukin; ILC3—type 3 innate lymphoid cells; DCs—dendritic cells; Treg cells—regulatory T cells; Th—T helper cells: LPS—lipopolysaccharide; IgA—immunoglobulin A.

Mucosal surfaces, particularly epithelia of the oral cavity and the gastrointestinal tract, are constantly interacting with a variety of microorganisms. These surfaces are also instrumental in metabolizing and absorbing food compounds and metabolites. While bacteria and metabolites are important for atherosclerosis development, proper epithelial function is essential for spatial and functional regulation of the host–microbe–metabolite(s) triad. The organization and functional regulation of epithelial barriers in the intestine and the oral cavity are governed by similar mechanisms and sets of rules (Figure 1a,b, left panel).

As the intestine is the most microbe-rich organ, its epithelium represents a major barrier that prevents translocation of luminal content, such as commensal and pathogenic bacteria, bacterial metabolites, and foreign antigens, into the systemic circulation [6]. Due to the constant exposure to trillions of bacteria, the intestinal surface has multiple lines of defense that include a mucus layer, epithelial cell barriers and junctions, and resident and infiltrating immune cells.

The mucus layer represents an efficient physical barrier that limits bacterial penetration into the epithelium. It contains mucins and other mucus structural proteins produced by goblet cells, various antimicrobial peptides, and immunoglobulin (Ig)A [87,88]. In the small intestine, where the bacteria burden is much lower, mucus consists of one layer that is not attached to the epithelium. In the large intestine, the mucus layer is thicker and consists of two parts: an inner layer that is attached to the epithelium and a loose outer layer trapping almost all bacteria. The gradient of antibacterial peptides within the mucus layer prevents the transition of microorganisms toward epithelial cells [88]. The knockout of bactericidal C-type lectin-regenerating islet-derived protein 3 gamma (RegIIIγ, encoded by the *Reg3g* gene) in mice resulted in an increased number of mucosa-associated bacteria [89,90]. IgA binds to bacteria, reducing their mobility in the mucus [88,91]. Mice deficient in the polymeric immunoglobulin receptor (*Pigr*^−/−^), which facilitates IgA transport into the lumen, are characterized by reduced luminal IgA and altered gut microbiota, promoting higher susceptibility to dextran sodium sulfate (DSS)-induce colitis [92]. Similarly, a deficiency in *Muc2*, a major component of mucus, leads to increased bacterial translocation and spontaneous intestinal inflammation [93].

The intestinal epithelium provides physical separation of luminal content from subepithelial sites via the formation of tight and adherens junctions. Lowered expression of tight junction proteins (TJPs), such as claudins, occludins, tricellulin, and junctional adhesion molecules (JAMs), increases intestinal permeability and allows bacteria and bacterial product translocation [94].

A large number of immune cells, including cells of innate and adaptive immunity, residing within the intestinal wall are specialized in the recognition and killing of bacteria that are able to escape the first two lines of defense [3]. These cells produce cytokines, which regulate antimicrobial immune responses, enhance or disrupt epithelial cell function, and could contribute to the development of atherosclerosis, acting locally in the gut or systemically (Figure 1a).

The oral cavity is covered by stratified squamous epithelia, which can be divided into three categories, depending on the function: masticatory mucosa, a protective keratin layer covering areas exposed by mechanical injury from chewing; specialized mucosa, involved in taste perception; and lining mucosa, a non-keratinized epithelium that allows direct interaction with microbes and other environmental agents [95,96,97]. The most vulnerable site of the oral cavity appears to be the tissue surrounding the teeth, known as gingiva. The mucosa in this area is in close proximity to the rich and diverse tooth-adherent microbial community, which often forms complex biofilms [97]. The translocation of oral microbes was reported after simple chewing or tooth brushing [98], suggesting this area as the entry site for oral bacteria. Saliva represents a rather unique protective antibacterial effector mechanism in the oral cavity and contains various antimicrobial components, including defensins, lysozyme, and lactoferricin, as well as secretory IgA capable of modulating oral microbiota [99]. A major triad of epithelial barriers described above that consists of mucus, an epithelial cell barrier and junctions, and epithelial immune cells holds true for the oral cavity as well. Just like for intestinal microbiome studies, the oral microbiome has been shown to contribute to various chronic inflammatory diseases such as colon cancer, IBD, rheumatoid arthritis, and, importantly, atherosclerosis [100,101,102,103,104,105]. Changes in the composition of oral microbiota or biofilm localization closer to epithelial cells and its increased translocation along with gingival crevicular fluid (GCF), a serum exudate that contains various host immune factors, such as complement components, immunoglobulins, and cytokines, were shown to contribute to the activation of immune cells that are abundantly present in the oral mucosa [106]. 

Neutrophils represent a majority (~95%) of cells found at the healthy gingival barrier, where they continuously transmigrate through the junctional epithelium and play a crucial role in the maintenance of mucosal homeostasis [96]. While research connecting alterations of the oral microbiome to atherosclerosis is still rather limited, it is quite possible that many cytokines involved in the control of inflammation in the intestinal wall would be implicated in host defense in the oral cavity. Therefore, the role of cytokines as gatekeepers at barrier tissues would likely impact systemic and distal inflammatory reactions and atherosclerosis development (Figure 1b).

## 4. Microbiota in CVD Development

The connection between unhealthy diet, alteration in the bacterial composition in the intestine and in the oral cavity, and cardiovascular disease development has recently emerged [107]. Since metabolic alterations contribute to atherosclerosis, possible contributions of gut microbiome to the disease development via altered production of microbial-derived or food-derived and microbially modified metabolites have been found and actively studied [108,109,110,111,112]. The potential effect of microbial recognition and microbial metabolites on the induction and maintenance of atherosclerosis-promoting chronic inflammation is likely to play an important role but currently is under-investigated. Microbial and viral components of commensal and pathogenic flora, collectively called microbiome, are key regulators of cytokine production and the differentiation of cytokine-producing cells. Even subtle quantitative and qualitative changes in the microbiome may be associated with the development of various inflammatory diseases, including colitis, obesity, and cardiovascular diseases [113,114]. Thus, changes in the gut microbiota or dysbiosis, caused by diet, stress, or the use of antibiotics, can have a significant impact on the local and systemic tuning of inflammation and on the development of chronic inflammatory diseases, including atherosclerosis. The altered microbiota can induce low-grade systemic inflammation by releasing microbiome-derived pro-inflammatory metabolites to the circulation, which, in turn, activate immune cells to produce pro-inflammatory mediators. Likewise, such changes in the microbiota along with genetic and epigenetic changes in inflammatory pathways may account for the interpersonal differences in the duration, magnitude, flavor, and outcome of inflammatory signaling (Figure 1).

Bacteria are capable to produce various metabolites that could be involved in a variety of metabolic pathways. Moreover, bacteria can be closely associated with host tissues or invade host tissues, along with the expression and presentation of pathogen-associated molecular patterns (PAMP) that are recognized by the host immune and inflammatory system. Therefore, mere changes in the amount, representation, quantity, and quality of the microbiome may account for changes in the host inflammatory response and disease development. Alternatively or in addition, changes in the properties or quality of barrier functions of the host at the epithelial surfaces can be decisive factors for why two similar microbiomes can have different interactions with host cells and the inner environment, resulting in differential regulation of the inflammatory response and disease manifestations. These two non-mutually exclusive possibilities are further discussed below.

The development and alteration of the microbiome composition may be influenced by different factors, including diet, lifestyle, environment, and antibiotic consumption. The gut microbiome is primarily composed of five major phyla: *Firmicutes*, *Bacteroidetes*, *Actinobacteria*, *Proteobacteria*, and *Cerrucomicrobia* [115]. Anaerobic bacteria taxonomies such as *Firmicutes* and *Bacteroidetes* are well established as contributing more than 90% of the total healthy intestinal microbiome composition. A high-protein diet increases the abundance of *Bacteroides* and reduces *Prevotella* [116], while a high-carbohydrate diet is conversely associated with the enrichment of *Prevotella* and lowered abundance of *Bacteroides*. High-fat diet (HFD) consumption leads to a decrease in *Bacteroidetes* abundance and increases both *Firmicutes and Proteobacteria* [117]. In addition, it has been shown that HFD, together with the antibiotic streptomycin, alters the energy metabolism of intestinal epithelial cells, increasing oxygen bioavailability, which favors the expansion of facultative anaerobes, for example, *Enterobacteriaceae*, associated with low-grade mucosal inflammation in the intestine [118]. Moreover, an HFD diet is able to alter gut barrier function through the reduction in tight junction proteins [119], causing changes in gut permeability and increasing the ability of bacteria-derived products to translocate into the systemic circulation, leading to chronic inflammatory disease development, i.e., atherosclerosis. Indeed, elevated serum lipopolysaccharide (LPS) levels were observed in high-fat-diet-fed mice [120]. Conversely, a high-fiber diet regulates the gut microbiome composition, leading to high prevalence of *Bacteroides acidifaciens* that can generate short-chain fatty acids (SCFAs), known to play a protective role in CVD development [111].

### 4.1. Intestinal Microbiota in CVD Development

Studies addressing the connection between pro-inflammatory gut microbiota and atherosclerosis development using the 16S rRNA gene sequencing approach revealed a significant reduction in several taxonomies, including *Akkermansia*, *Christensenellaceae*, *Clostridium*, and *Odoribacter*, in the gut microbiota of mice with atherosclerosis. High abundance of *Lactobacillus reuteri* was associated with elevated high-density lipoproteins (HDL). *Bifidobacterium* bacteria are known for their anti-inflammatory effects and have been used as probiotics assisting mucosal barrier function and reducing systemic inflammation by controlling intestinal LPS level [121].

Alterations in the bacterial taxa balance associated with pro-inflammatory changes and CVD progression could contribute to the expansion of bad bacteria that simply overcomplete with good bacteria for an ecological niche in more favorable conditions driven by diet or antibiotics [118,122]. Therefore diet, antibiotics, or other environment-driven changes in intestinal epithelial cell function affecting the microbiota composition may cause the ablation of protective commensals, allowing the expansion of bacteria with more pathogenic properties, for example, those bacteria that are capable of mucus degradation, thereby reducing barrier protective function [118,123].

Moreover, the components of the microbiota can directly activate immune cells situated at barrier tissues. An altered gut mucosal layer and increased permeability combined with adhesiveness of bacteria are likely to contribute to the translocation of endotoxins to the systemic circulation [71]. The recognition of lipopolysaccharide (LPS), the major component of the outer membrane of Gram-negative bacteria, links gut and oral microbiome alterations to cardiovascular diseases [124,125,126]. Increased association of LPS with the intestinal epithelium is capable of inducing low-grade systemic inflammation and thus cardiovascular disease progression through Toll-like receptor (TLR)-4-driven pro-inflammatory cytokine and chemokine expression [127], including IFNγ, IL-1, IL-6, IL-8, and TNFα. LPS can promote the expression of adipocyte enhancer-binding protein 1 (AEBP1) [128], scavenger receptor CD36 [129], and adipose differentiation-related protein (ADRP) [130], which mediate lipid accumulation in macrophages.

In addition, bacteria-derived metabolites may directly regulate immune cell activation and function. For instance, the gut microbiome composition in patients with coronary artery disease (CAD) is characterized by increased abundance of several bacterial taxa, including *Roseburia*, *Klebsiella*, *Clostridium IV*, and *Ruminococcaceae*, that could be involved in taurine, sphingolipid, ceramide, and benzene metabolism [131]. These correlations suggest a host–microbiota interplay implicated in the inflammation in atherosclerosis. Additionally, some commensal bacterial species in the gut, such as *Lactobacillus paracasei* and *Escherichia coli*, signal to intestinal enterocytes to regulate their lipid metabolism [109]. *L. paracasei* controls the production of L-lactate, which upon conversion to malonyl-coenzyme A (CoA), inhibits beta-oxidation of lipids, including oxidation of lipoproteins and their further uptake by macrophages during foam cell formation [109]. *E. coli* inhibits chylomicron secretion by enterocytes but, in contrast to *L. paracasei*, promotes lipid oxidation by transcriptional control of enzymes involved in lipid oxidation, therefore contributing to foam cell formation [109]. The relatively low abundance of *Eubacterium* and *Roseburia*, along with the enriched presence of the *Collinsella* genus, was found in the gut microbiome of patients with symptomatic atherosclerosis, compared to healthy controls, suggesting their potential pathogenic role. Metagenomic analysis of samples enriched in *Collinsella* spp. revealed an increased abundance of genes encoding members of the peptidoglycan biosynthesis pathway that could prime cells of the innate immune system, thereby driving the progression of atherosclerosis [132].

Multiple bacteria, including species of *Clostridium*, *Desulfitobacterium*, *Enterococcus*, *Streptococcus*, *Anaerococcus*, *Klebsiella*, *Pseudomonas*, *Desulfovibrio*, *Citrobacter*, and *Enterobacter*, facilitate the biosynthesis of trimethylamine (TMA) from dietary choline and carnitine [133]. TMA is a metabolic precursor that is converted to a trimethylamine N-oxide (TMAO) in the liver. TMAO drives atherosclerosis progression [110], in part, by regulating macrophage activation and foam cell formation, platelet activation, and aggregation, resulting in elevated expression of IL-1β, IL-6, and TNFα inflammatory cytokines [134]. Recent work implies that TMAO could be also involved in metabolic dysfunction and activation of inflammation, directly acting via its receptor protein kinase R (PKR)-like endoplasmic reticulum kinase (PERK) [135]. An increased abundance of *Akkermansia*, *Prevotella*, and *Ruminococcus* gnavus in the intestine was associated with higher serum TMAO levels during coronary artery disease development [136]. By now, at least several bacterial species, including *Anaerococcus hydrogenalis*, *Proteus penneri*, *Providencia rettgeri*, *Clostridium asparagiforme*, *C. hathewayi*, *C. sporogenes*, *Esherichia fergusonii*, and *Edwardsiella tarda*, were directly implicated in TMA generation [137]. Likely many more bacteria, which contain the biosynthetic apparatus for TMA synthesis (TMA lyase gene), are capable of producing TMA in a physiologically relevant context and quantities [136,138].

Commensal microbiota, including *Akkermansia*, *Christensenellaceae*, *Clostridium*, *and Odoribacter*, are also capable of fermentation of food-derived fibers to produce short-chain fatty acids (SCFAs), most notably butyrate, propionate, and acetate [139]. Short-chain fatty acids possess anti-inflammatory properties by regulating macrophages and T regulatory cells, thereby reducing pro-inflammatory cytokine production [108,140,141]. Butyrate has been shown to play an anti-inflammatory role in atherosclerosis via inhibition of the endothelial cell migration and proliferation by acting through free fatty acid receptor 3 (Ffar3) [142]. In addition, SCFAs, particularly butyrate, were shown to act on intestinal epithelial cells, regulating their metabolism and facilitating proper healthy barriers, including tight junction protein production, thereby preventing excessive signals from the commensal microbiota [143]. Indeed, in general, SCFA- and butyrate-producing bacteria were shown to inversely correlate with various CVD severity, including atherosclerosis [144]. For example, the abundance of key butyrate-producing bacteria *Roseburia intestinalis* and *Faecalibacterium prausnitzii* decreased in the gut microbiome of atherosclerotic patients, while enrichment in *Enterobacteriaceae (E. coli*, *Klebsiella* spp., and *Enterobacter aerogenes*), *Streptococcus spp.*, *Lactobacillus salivarus*, *Solobacterium moorei*, *Atopobium parvulum*, *Ruminococcus gnavus*, and *Eggerthella lenta* was observed [144]. Further work also supported an inverse correlation of *Roseburia intestinalis* with atherosclerosis development due to butyrate-producing capability and demonstrated that intestinal administration of butyrate reduced the systemic endotoxemia and atherosclerosis in a mouse model [145]. While SCFAs clearly inhibit inflammatory pathways locally and systemically, it remains to be determined experimentally whether they are chief protectors against atherosclerosis and whether they act solely though their anti-inflammatory actions on immune cells or act via other metabolism-related pathways systemically or locally inside the plaque.

Despite numerous reports discovering a correlation between particular bacterial families and inflammatory disease development, the specific and exclusive causative role of particular bacterial taxonomies (aka the Koch postulate for infectious diseases) is difficult to demonstrate in all patients or in all animal models housed at different facilities to unequivocally prove such a mechanistic link.

In addition, a number of studies looking at the correlation between particular bacteria and atherosclerosis development suggested potential ambiguity in the specific roles of particular bacteria [146]. For example, members of *Lachnospiraceae* family are known to be one of the main producers of atheroprotective and anti-inflammatory SCFAs, but at the same time, increased *Lachnospiraceae* abundance, particularly the *Dorea* genus, in the gut led to increased mucin degradation, thereby reducing barrier function and potentially enhancing inflammation originating from the gut [146]. The presence of greater numbers of bacteria from the *Lachnospiraceae* family was also associated with a reduced mucus layer and an increased serum level of bacteria-derived metabolites with pro-atherogenic properties, and correlated with accelerated atherosclerosis [71]. The ambiguous role of *Akkermansia muciniphila* in inflammatory diseases has also been described [147,148,149]. These bacteria are known degraders of mucus [148] but also potent producers of anti-atherogenic SCFAs (propionate and acetate) [149], and overall, their higher abundance was correlated with enhanced atherosclerosis in a mouse model, with the possible mechanism being the facilitation of systemic translocation of microbiome-derived metabolites [71]. The presence of the *Prevotella* genus is generally considered to be pro-inflammatory since these bacteria drive Toll-like receptor (TLR)-2-dependent cytokine induction and increased activation of the T helper IL-17-producing cell (Th17) response in a variety of intestinal inflammation models, where it contributes to both barrier deterioration and low-grade systemic inflammation [150]. *Prevotella* is a TMAO producer, and its presence is associated with a higher risk of thrombosis [110] and increased concentrations of low-density lipoproteins (LDL) in the blood of patients with cardiac valve calcification [151]. However, characterization of healthy human intestinal microbiome composition has revealed a prevalence of *Prevotella* in the mucosa of oral and gut ecosystems, and the abundance of the *Prevotella* genus was shown to be inversely correlated with CVD risk factors, including blood pressure and triglycerides [152]. Perhaps, contradicting observations regarding *Prevotella* spp. and CVD are related to the fact that numerous bacteria species within *Prevotella* genus potentially contain different metabolic genes and play different, not yet identified, specific roles in the regulation of inflammation.

It becomes, therefore, evident that certain gut microbiome alterations are associated with increased local and systemic inflammation, contributing to atherosclerosis progression. This likely includes multiple potential mechanisms, including changes in microbial metabolites, intestinal wall permeability, and defects in the protective mucus layer, leading to local, systemic, or distant (in atherosclerotic plaque) activation of immune and non-immune cells. Overall, it is tempting to suggest that a specific functional characteristic of bacteria, particularly their ability to regulate the production of certain metabolites rather than their species identity, is more important for the outcomes of inflammatory diseases, including atherosclerosis.

### 4.2. Oral Microbiota in CVD Development

The oral cavity represents yet another major site for microbiota colonization and robust host–microbiota interactions, affecting local and systemic inflammatory responses. The microorganisms found in the oral cavity constitute the second-largest and diverse microbial community after the gut [153]. A high abundance of pathogens in the oral cavity in inflammatory diseases, such as periodontal disease, could potentially contribute to low-grade systemic inflammation and thus to atherosclerosis. A significant difference in the oral microbiome composition was demonstrated between patients with atherosclerosis and a control cohort, with a higher relative abundance of the bacterial taxa *Anaeroglobus* correlated with the disease [154]. Additionally, the oral cavity microbiome of symptomatic atherosclerosis patients exhibited significant enrichment of *Lactobacillus*, *Capnocytophaga*, and *Catonella*, whose abundance further correlated with inflammatory and atherosclerosis-related markers such as C-reactive protein (CRP) and apolipoproteins [100]. Microbial communities from the oral cavity of atherosclerotic patients revealed the abundance of *Fusobacterium*, which positively correlated with LDL and total cholesterol, while the abundance of *Streptococcus* correlated with atheroprotective HDL and apolipoprotein A1 (ApoAI) [154], suggesting that the oral microbiome could contribute to atherosclerotic disease, for instance, by regulating host lipid metabolism. Further studies also identified *Chryseomonas* and *Veillonella* in the oral cavity of patients with atherosclerosis, but their functional relevance and mechanisms of their action remain to be determined [154].

Periodontitis is a chronic inflammatory disease that is characterized by elevated production of several pro-inflammatory cytokines, including IL-1β, IL-6, and TNFα, in the oral cavity, which can further contribute to low-grade systemic inflammation. The link between periodontitis and cardiovascular disease development was suggested by several studies [155], and therapy against periodontitis was shown to substantially decrease the level of pro-inflammatory CRP in patients with atherosclerotic cardiovascular disease [156]. *Prevotella* spp., particularly *Prevotella nigrescens*, were proposed to link oral microbiome dysbiosis and atherosclerotic disease progression based on higher abundance of these bacteria in subgingival plaques [105]. The oral pathogen *Porphyromonas gingivalis* increased atherosclerosis progression in *Apoe*^−/−^ mice compared to uninfected controls, promoting the activation of T cells and macrophages and an increase in the total cholesterol level [102]. Interestingly, the accumulation of immune cells in atherosclerotic plaques was prevented by immunization with heat-killed *Porphyromonas gingivalis* prior to pathogen exposure, indicating a possibility to induce a tolerogenic response to the pro-atherogenic microbiota [102]. Additionally, the protective role of TLR4 signaling was reported in response to infection with the oral pathogen *Porphyromonas gingivalis*, since TLR4-deficient *Apoe*^−/−^ mice demonstrated enhanced atherosclerotic lesion progression and impaired Th1 and regulatory T cell (Tregs) infiltration compared to control mice [101]. Overall, oral microbiota alterations influence inflammation and atherosclerosis development.

## 5. How Cytokines Regulate Barriers and Change Host–Microbiota Interactions

Altered cytokine signaling is involved in the control of epithelial cells at mucosal surfaces. On the other hand, aberrant epithelial cell activity results in changes in microbiota composition and function and directly impacts immune cell activation, inflammation, and atherosclerosis. Cytokines regulate intestinal homeostasis through multiple mechanisms. While most of the observations discussed below are derived from models of cytokine contribution to different inflammatory diseases, such as colitis, similar mechanisms are likely to be implicated to the pathogenesis of atherosclerosis. For instance, increased gut permeability with reduced tight junction protein expression in the intestinal tissue in rats was associated with hypertension [157], a known risk factor for atherosclerosis development. Moreover, attenuation of Western-diet-induced intestinal permeability by non-absorbable antibiotics (neomycin and polymyxin) or curcumin supplementation ameliorated atherosclerosis development in *Ldlr*^−/−^ mice [158]. *Apoe*^−/−^ mice with knockdown of intestinal fatty-acid-binding protein (*Fabp2*) demonstrated decreased atherosclerotic lesion development associated with improved intestinal permeability and enhanced zonula occludens-1 (ZO-1) and occludin tight junction protein expression [159].

Moreover, chronic inflammatory conditions in the gut or in the oral cavity themselves are important predisposing factors for atherosclerosis [155,160]. Increased risk of cardiovascular events accompanied by elevated expression of early atherosclerosis markers has been shown in patients with inflammatory bowel disease [160,161,162].

### 5.1. Cytokines of the IL-1 Family in Barrier Tissue Inflammation

In the context of intestinal homeostasis, IL-1 family members demonstrate both protective and pathogenic properties. For instance, IL-1R signaling was shown to drive intestinal inflammation in DSS-induced colitis, as mice deficient in the single immunoglobulin IL-1 receptor-related IL-1 pathway inhibitory molecule (SIGIRR, also known as TIR8) [163] were more susceptible to DSS-induced colitis compared to controls [164,165]. Mice deficient in *Il1a* were more resistant to DSS colitis, whereas *Il1b*^−/−^ mice had more severe colitis and impaired tissue repair capacity compared to wild-type (WT) mice [166]. Specific ablation of IL-1α in intestinal epithelial cells (IECs) led to a similar colitis phenotype as global IL-1α knockout, suggesting that IEC-derived IL-1α drives the inflammatory response in the intestine during DSS-induced colitis, whereas IL-1β has a protective role and involved in tissue repair [166]. A recent study revealed that the resistance of *Il1a*^−/−^ to DSS colitis is microbiota dependent since co-housing of these mice with WT controls diminished their disease resistance [167]. On the other hand, caspase-1 (a key inflammasome component) deficiency resulted in limited IL-1β and IL-18 production associated with ameliorated inflammation in DSS-induced colitis [168]. Moreover, antibody neutralization of IL-1β protected mice from *Helicobacter hepaticus*-triggered colitis by limiting the accumulation of granulocytes and IL-17A-producing innate lymphoid cells [169].

IL-18/IL-18R signaling in intestinal epithelial cells was also shown to drive DSS colitis in mice, whereas IL-18BP protected mice from colitis, suggesting a colitogenic function of IL-18 [170]. As mentioned above, various environmental factors, including diet and frequent antibiotic use, impact the commensal microbiota composition, which, in turn, affects the expression of cytokines in the intestine. It was demonstrated that the magnitude of induction of cytokines, for example, IL-23, IL-17, IL-22, or IL-1, can be regulated by specific microbes, or by the bulk commensal microbiota, which invade host tissues as a result of deteriorated barrier function [171,172].

The presence of pathogens in the gut is detected by numerous pattern recognition receptors (PRRs), including Toll-like receptors (TLRs) and Nod-like receptors (NLRs) [173]. This process results in the activation of a cytokine response that is crucial for host defense. Activation of NLRs leads to the formation of inflammasomes and maturation of IL-1β and IL-18 cytokines [174], which play an important role in host defense. IL-1 and IL-18 have been shown to protect mice during *C. rodentium* infection and therefore prevent pathogen-induced systemic inflammation. IL-1R ablation resulted in higher susceptibility of mice to *C. rodentium*-driven intestinal pathology and increased intestinal permeability [175]. Mice lacking the inflammasome components *Nlrp3*, *Nlrc4*, caspase-1, IL-1β, and IL-18 were also characterized by impaired clearance from *C. rodentium* [176]. Microbiota-derived metabolites promote NLRP6-dependent IL-18 production in the intestine, which, in turn, regulates colonic antimicrobial peptide expression. IL-18 production was induced by taurine, a metabolite enriched in WT mice, whereas dysbiotic-microbiota-associated metabolites histamine and spermine negatively regulated IL-18 expression [31].

In the oral cavity, IL-1 was shown to regulate the Th17 response during infection with *Candida albicans* but not in healthy or periodontitis (inflamed) conditions [95]. *C. albicans* is able to form invasive filamentous hyphae that secrete a pore-forming toxin called candidalysin, which damages oral epithelial cells and triggers the activation of the immune response and IL-1 cytokine production [177]. Candidalysin-driven IL-1α/β production, in turn, drives the proliferation of innate IL-17^+^TCRαβ^+^ cells and the expression of IL-17 needed to combat the infection [178]. One could speculate that pathogen-driven differentiation of T cells to Th17 in the oral cavity might supply the pool of pathogenic Th17 cells that could potentially contribute to inflammation in atherosclerotic plaque and elsewhere, in part, by promoting neutrophil recruitment in IL-17A-dependent manner. Indeed, *Apoe*^−/−^ mice fed with HFD and inoculated with a periodontal pathogen *Porphyromonas gingivalis* demonstrated an increase in atherosclerosis, while the ablation of pathogen-induced IL-1R signaling ameliorated the disease [14].

IL-18 and IL-36, other members of the IL-1 family, also regulate oral immunity to fungi, such as *Candida*, or to bacteria, such as *Porphyromonas gingivalis* [179,180,181]. Elevated levels of IL-1β and IL-18 were detected in the gingival crevicular fluid (GCF), saliva, and serum of patients with periodontitis [182,183,184,185]. These observations suggest a mechanistic link between periodontitis and gum inflammation and atherosclerosis.

### 5.2. Cytokines of the IL-17 Family in Barrier Tissue Inflammation

The Th17 immune response and the IL-23–IL-17A axis have been shown to be involved in the pathogenesis of various autoimmune diseases, including IBD [83]. Since IL-17A was shown to induce pro-inflammatory gene expression, it was expected to promote intestinal pathology. However, clinical trials utilizing anti-IL17A monoclonal antibodies (secukinumab) for Crohn’s disease treatment demonstrated that the blockade of IL-17A was ineffective and, moreover, resulted in increased frequency of fungal infections, suggesting the protective function of IL-17A in the context of intestinal immunity [186]. Indeed, *Il17a*^−/−^ mice demonstrated more severe inflammation in DSS-induced colitis compared to controls, which was associated with increased intestinal permeability [187]. Furthermore, antibody-mediated neutralization of IL-17RA or IL-17A led to enhanced colitis in *Abcb1a*^−/−^ mouse model (*Abcb1a* gene in mice encodes the P-glycoprotein-ATP-dependent transporter) that was associated with increased intestinal permeability and reduction in antimicrobial peptide β-defensin, RegIIIγ, and S100A8 expression [188]. IL-17A has been shown to be involved in host defense during *Salmonella typhimurium* infection via regulation of CXCL1-dependent neutrophil recruitment and antimicrobial peptide expression; and *Il17ra*^−/−^ mice demonstrated higher susceptibility to *Salmonella* and *Citrobacter rodentium* infection, associated with decreased expression of β-defensins and intestinal IgA responses [37,189,190]. The commensal microbiota, including segmented filamentous bacteria (SFB), regulates the development of IL-17A-producing Th17 cells [171]; conversely, IL-17A signaling also controls the intestinal microbiome. Intestinal epithelial-cell-specific knockout of *Il17ra* or *Il17rc* (*Il17ra/Il17rc^flox/flox^Villin-Cre* mice) promoted colonization of mice by segmented filamentous bacteria (SFB), which was associated with reduced expression of IL-17A target genes such as α-Defensin, *Nox1*, and *Pigr* [191]. Moreover, mice with specific inactivation of *Il17ra* in intestinal epithelial cells presented with dysbiosis and increased CpG-rich DNA translocation into the liver, resulting in enhanced liver IL-18 production and aggravation of hepatitis in the concanavalin A (ConA) model of T-cell-mediated hepatitis [192]. This altogether established IL-17 signaling as a major regulator of resistance to microbes, which is essential for the regulation of host–microbiota interaction and microbial product translocation. A protective role of IL-17 signaling in the intestine could explain the discrepancy in conclusions regarding the role of IL-17 in atherosclerosis using global but not cell-type-specific genetic or pharmacological manipulations in mouse models, where both pathogenic and protective functions of this cytokine could be neutralized.

In contrast to other mucosal sites, such as the gut, skin, and eye, where the development of Th17 cells is microbiota-dependent, in the gingival barrier Th17 cells were surprisingly detected even in germ-free mice, indicating innate requirements for these cells and the cytokines they produce. During fungal infection, IL-17 is expressed by different cell types, including γδT cells, ILC3, and innate-acting population of CD4^+^ natural IL-17-producing T cells (nTh17) [178,193,194], where only nTh17 cells expand in the oral cavity in an IL-1- but not IL-6- or IL-23-dependent manner [178]. Several studies have discovered that deficiency of IL-17RA, IL-17RC, IL-17F, and *Act1*, an essential component of IL-17 signaling, is associated with chronic mucocutaneous candidiasis in patients, suggesting a protective role of the IL-17 response [195,196,197]. Interestingly, mice with genetic ablation of IL-17RA or IL-17RC or pharmacological inhibition of IL-17A or IL-17F demonstrated normal neutrophil recruitment to the tongue after *C. albicans* infection but still failed to clear the fungus [198], whereas others showed that *Il17ra*^−/−^ mice, but surprisingly not mice with oral epithelial-cell-specific deletion of IL-17RA, had impaired recruitment of neutrophils [199,200]. IL-17 is also known to induce antimicrobial peptide expression in response to infection [198,199,200]. For example, IL-17-driven expression of S100A9 and β-defensins has been demonstrated in *C. albicans* infection [198,199,200].

While the IL-17/Th17 pathway is protective against *C. albicans* infection, it seems to play a pathogenic role in periodontitis [201,202], implying a context-specific role of this cytokine pathway. Recently, it was shown that periodontitis-associated pathogens induce the development of IL-17-producing Th17 cells, which can then transmigrate to the gut and contribute to colitis development in mice [103]. It could be possible that these activated Th17 cells could also migrate to the vessel wall and contribute to the inflammation in atherosclerotic plaque.

### 5.3. Cytokines of the IL-10 Family in Barrier Tissue Inflammation

IL-10 is produced by various immune cells and considered as a major anti-inflammatory cytokine that plays an essential role in intestinal inflammation. Mice deficient in both IL-10 and IL-10R spontaneously develop colitis [203] as IL-10 suppresses the inflammatory activity of various immune cells, and colitis in *Il10*^−/−^ mice is mediated by Th1 cells and IFNγ [204]. CX3CR1^+^ macrophage-derived IL-10 is not required to tame colitis in mice; however, IL-10R expressed by macrophages is essential to prevent fulminant enterocolitis with upregulated pro-inflammatory genes, including *Nos2*, *Il23a*, *Ccl5*, *Ccr7*, and *Saa3* [205]. A later study revealed that IL-10R-deficient CX3CR1^+^ macrophages are characterized by elevated expression of IL-23 that induces IL-22 secretion by Th17 and ILC3, suggesting the role of the IL-23–IL-22 axis in intestinal pathology [206]. Indeed, double inactivation of both IL-10R and IL-23 in CX3CR1 macrophages (*Il10ra^flox/flox^Il23a^flox/flox^ Cx3cr1-Cre* mice) protected mice from colitis development, and moreover, *Il10ra^flox/flox^ Cx3cr1-Cre* mice lacking IL-22 (*Il10ra^flox/flox^ Cx3cr1-Cre Il22^−/−^* mice) did not develop colitis [206]. These observations suggest an important cytokine network crucial for the development of inflammation at the intestinal barrier. The role of IL-10 in host defense in the oral cavity is poorly understood. A recent study demonstrated that Treg cell depletion as well as genetic ablation of IL-10 in mice did not affect *Candida albicans* colonization in the oral mucosa [207]. In the case of periodontitis, increased accumulation of Tregs capable of producing IL-10 [208,209] suppressed the inflammation in periodontal tissue. Indeed, *Il10^−/−^* mice demonstrated high susceptibility to bone loss induced by the periodontal pathogen *P. gingivalis* [210] and ligature-induced periodontitis [211]. Taken together, IL-10 is uniformly anti-inflammatory in the context of atherosclerosis, the intestine, or the oral cavity. IL-10 anti-inflammatory action at mucosal surfaces can potentially prevent overt autoimmunity and translocation of microbial metabolites into systemic circulation.

A plethora of studies provides strong evidence that IL-22 is an essential cytokine for host defense at the intestinal mucosa, most notably by its known role in the control of proliferation and regeneration of intestinal epithelial and goblet cells, expression of RegIIIβ, RegIIIγ, S100A8, and S100A9 antimicrobial peptides [212,213,214,215,216]. These functions of IL-22 were uniform for the conditions of infections by *Salmonella*, *Citrobacter*, or *Candida*, different models of intestinal inflammation or HFD-induced intestinal barrier alterations [217]. Administration of recombinant IL-22 to ILC-depleted mice prevented peripheral dissemination of commensal bacteria caused by depletion of ILC and reduced expression of antimicrobial peptides RegIIIβ and RegIIIγ that was restored after IL-22 administration [212]. IL-22 expression can be regulated by microbiota-derived ligands such as indole-3-acetic acid (IAA), produced from dietary tryptophan by colonic bacteria, stimulating the aryl hydrocarbon receptor (AHR) [218]. It has been shown that in a mouse model of alcoholic liver disease, ethanol-induced dysbiosis results in reduced intestinal levels of IAA, and that, in turn, decreases IL-22 production by ILC3 and RegIIIγ expression, leading to bacterial translocation into the liver, causing excessive liver damage [218].

A recent study revealed that mice lacking IL-22RA1 or the signal transducer and activator of transcription 3 (STAT3) in the oral basal epithelial layer demonstrate susceptibility to oropharyngeal candidiasis. Moreover, IL-22/STAT3 signaling is involved in epithelial cell proliferation and survival during oropharyngeal candidiasis and in restoring the ability of the oral epithelium to respond to IL-17A [219]. In the context of atherosclerosis, IL-22 deficiency is linked to lowered expression of RegIIIβ and RegIIIγ antimicrobial peptides, altered barrier function, and elevated serum LPS levels, therefore positioning IL-22 as an important player at the intestinal barrier needed to protect from the disease development. Further work addressing the cell-specific role of IL-22R will be needed for a better understanding of this cytokine function in atherosclerosis.

### 5.4. Cytokines of the IL-6/IL-12 Superfamily in Barrier Tissue Inflammation

IL-6 is produced by lamina propria myeloid cells in the gut and is known for its pro-inflammatory effects inducing pro-inflammatory gene expression by target cells [220]. However, IL-6 is also involved in intestinal homeostasis and has been shown to protect mice from DSS-induced colitis [221,222] and promote epithelial regeneration and barrier function by regulating tight junction protein Claudin 1 expression and thickness of the mucus layer [223,224]. On the other hand, IL-6, as an acute phase protein, is essential for regulation of antibacterial immune responses [220]. These observations, together with the type of IL-6R signaling, will likely explain the discrepancy in the results obtained in models of atherosclerosis using mice with global IL-6 deficiency.

Early studies have suggested that IL-12 is involved in the regulation of intestinal and systemic inflammation and defense, since neutralization of IL-12p40 ameliorated colitis in mice [225,226], while IL-12p40 ablation led to enhanced susceptibility to intestinal infections [227,228,229]. As IL-12p40 is a common subunit for IL-12 and IL-23, some local effect on intestinal inflammation and barriers was mediated by IL-23, while systemic inflammation was primarily regulated by IL-12 [230,231,232]. IL-12 was shown to drive intestinal pathology in early stages of colitis in mice lacking the nuclear factor kB (NF-kB) essential modulator (NEMO, also named IKKγ) specifically in intestinal epithelial cells (IECs), (NEMO^IEC-KO^), whereas IL-23 became important during later stages of the disease [233].

IL-23 is also essential for host defense against infections and potential invasion of the commensal microbiota and metabolites translocation [213], with many of these functions enabled by the downstream action of IL-23-driven cytokines IL-22 and IL-17 or IL-36 [71,234,235]. Administration of recombinant IL-23 was able to promote bacterial clearance via induction of IL-22 secretion by ILC3 [236]. Interestingly, IL-23 could also act via intestinal epithelial cells, inducing the c-type lectin RegIIIβ-mediated recruitment of IL-22-producing cells, and in the absence of this signaling (*Il23r^flox/flox^Villin-Cre*), mice were susceptible to DSS colitis and were characterized by dysbiosis [237]. Hematopoietic and global IL-23 deficiency led to accelerated atherosclerosis, associated with lowered expression of RegIIIβ and RegIIIγ, and reduced levels of mucins, in turn, resulting in gut microbiota alterations with the expansion of pro-atherogenic bacteria [71]. IL-23, together with IL-6, is involved in the differentiation of pathogenic Th17 cells during periodontitis [95]. Although an increased level of IL-6 was detected in the GCF of chronic periodontitis patients [238], the role of this cytokine in host defense at the oral mucosa site requires further investigation. On the other hand, mice deficient in *Il12p40* were susceptible to oral *C. albicans* infection, but this effect was mediated mostly by IL-23/Th17 pathways rather than IL-12 [200]. Indeed, *Il23p19*^−/−^ mice demonstrated high susceptibility to oropharyngeal candidiasis, whereas *Il12p35*^−/−^ mice were characterized only by a slight increase in the fungal burden [200], implying that IL-23 but not IL-12 protects mice from oral candidiasis [200]. Interestingly, in the case of oropharyngeal candidiasis, IL-36 induced IL-23 seems to have separate effects from Th17/IL-17 pathway, which is induced by IL-1 [181]. Whether intestinal or periodontic inflammation heightened by a lack of proper cytokine-mediated control of the microbiota equally regulates atherosclerosis requires further investigation.

## 6. Concluding Remarks

It has become increasingly clear that alterations in intestinal or oral microbiota correlate with atherosclerosis development. Microbiota-derived metabolites affect the activation of immune cells abundantly present at mucosal surfaces, which, in turn, travel to distant sites, for example, to the arterial wall, to promote inflammation, which, together with other factors, for instance, elevated lipid accumulation, drives atherosclerosis. These metabolites can also diffuse systemically and activate immune cells inside the plaque or in other sites such as bone marrow and secondary lymphoid organs. Cytokine signaling plays an important role in various inflammatory processes, including the control of host–microbiome interactions at barrier tissues. Cytokine function at barrier tissues, in addition to their direct inflammatory role in the plaque, therefore represents an additional exciting mechanistic link between altered microbiota and CVD. Therefore, a better understanding of cell- and site-specific roles of cytokines would be necessary for efficient therapy development for cardiovascular diseases.

## Figures and Tables

**Table 1:** The role of selected cytokines in atherosclerosis.

Cytokine	Effect	Model	Reference
IL-1⍺, IL-1β	Promote atherosclerosis	- Hematopoietic deficiency of IL-1α in *Apoe*^−/−^ or *Ldlr*^−/−^ mice - Whole-body IL-1β knockout in *Apoe*^−/−^ mice - The anti-IL1β treatment of *Apoe*^−/−^ mice - Whole body IL-1R knockout in *Apoe*^−/−^ mice - Administration of soluble IL-1R antagonist (anakinra) in *Ldlr*^−/−^ mice - Smooth muscle cell-specific ablation of IL-1R in *Apoe*^−/−^ mice	[17,19] [20] [13] [14] [21] [18]
	Suppress atherosclerosis or have no effect	- Anti-IL-1β treatment during the late stages had no effect in *Apoe*^−/−^ mice	[18]
IL-6	Promote atherosclerosis	- Treatment of *Apoe*^−/−^ mice with recombinant IL-6 - Inhibition of IL-6 trans-signaling with soluble glycoprotein 130 (sgp130) in *Ldlr^−/−^* mice	[75] [79]
	Suppress atherosclerosis or have no effect	- Whole body IL-6 knockout in *Apoe*^−/−^ mice	[76,77]
IL-10	Promote atherosclerosis		
	Suppress atherosclerosis	- Whole body IL-10 knockout in *Apoe^−/−^* mice - Hematopoietic deficiency of IL-10 in *Ldlr^−/−^* mice - Systemic and local adenovirus-mediated suppression of IL-10 in *Ldlr^−/−^* mice - IL-10 overexpression by T cells in *Ldlr^−/−^* mice	[58] [59] [60] [61]
IL-12	Promote atherosclerosis	- Treatment of *Apoe*^−/−^ mice with recombinant IL-12 - Whole body IL-12 knockout in *Apoe*^−/−^ mice	[81] [82]
	Suppress atherosclerosis		
IL-17	Promote atherosclerosis	- Whole body IL-17A and IL-17RA knockout in *Apoe*^−/−^ mice - Neutralization of IL-17 in *Apoe*^−/−^ mice by monoclonal antibodies - Neutralization of IL-17 in *Apoe^−/−^* mice by adenovirus-encoded soluble IL-17RA inhibitor - Whole body IL-17C knockout in *Apoe*^−/−^ mice	[40] [43] [49] [50]
	Suppress atherosclerosis	- Whole body IL-17A knockout in *Apoe*^−/−^ mice - Neutralization of IL-17A in SOCS3-deficient *Ldlr*^−/−^ mice - Administration of recombinant IL-17 into *Ldlr*^−/−^ mice - Hematopoietic deficiency of *Trim21* in *Ldlr*^−/−^ mice - Neutralization of IL-17A in *Ldlr*^−/−^ mice transplanted with *CD4-Cre*^+^Smad7^fl/fl^ bone marrow	[42] [46] [46] [39] [44]
IL-18	Promote atherosclerosis	- Treatment of *Apoe*^−/−^ mice with recombinant IL-18 - IL-18 neutralization by IL-18-binding protein in *Apoe*^−/−^ mice - Whole body IL-18 knockout in *Apoe*^−/−^ mice - Treatment of SCID/*Apoe*^−/−^ mice with recombinant IL-18 - Whole body IL-18R knockout in *Apoe*^−/−^Ncc^−/−^ mice	[26] [27] [28] [29] [30]
	Suppress atherosclerosis		
IL-22	Promote atherosclerosis	- Whole body IL-22 knockout in *Apoe*^−/−^ mice	[70]
	Suppress atherosclerosis	- Hematopoietic deficiency of IL-22 in *Ldlr*^−/−^ mice - Administration of recombinant IL-22 into *Ldlr*^−/−^ mice	[71]
IL-23	Promote atherosclerosis	- Elevated level of IL-23 in CVD	[84]
	Suppress atherosclerosis	- Hematopoietic deficiency of IL-23 or IL-23R in *Ldlr*^−/−^ mice	[71]

CVD—cardiovascular disease; IL—interleukin; Apoe—apolipoprotein E; Ldlr—low-density lipoprotein receptor; SOCS3—suppressor of cytokine signaling 3; TRIM21—Tri-partite motif 2.
